# Veno-venous extracorporeal membrane oxygenation in managing acute respiratory distress syndrome associated with hemolytic uremic syndrome and septic shock: a case report

**DOI:** 10.1007/s10047-024-01457-9

**Published:** 2024-06-25

**Authors:** Genta Kinoshita, Asami Ito-Masui, Takafumi Kato, Fumito Okuno, Kaoru Ikejiri, Ken Ishikura, Kei Suzuki

**Affiliations:** 1 Emergency and Critical Care Center, Mie University Hospital, 2-174 Edobashi, Tsu City, Mie, 514-8507 Japan; 2 Department of Clinical Engineering, Mie University Hospital, Tsu City, Mie, Japan

**Keywords:** Extracorporeal membrane oxygenation (ECMO), Hemolytic uremic syndrome (HUS), Hemolysis

## Abstract

Veno-venous extracorporeal membrane oxygenation (VV-ECMO) is a rescue therapy for severe respiratory failure in which conventional mechanical ventilation therapy is unsuccessful. Hemolysis during VV-ECMO support arises from multiple factors associated with organ damage and poor outcomes. Therefore, close and prompt monitoring is needed. Hemolytic uremic syndrome (HUS) is characterized by hemolysis, acute renal failure, and thrombocytopenia. Hemolytic features of the disease may complicate VV-ECMO management. A 26-year-old man with a history of cerebral palsy underwent VV-ECMO for acute respiratory distress syndrome (ARDS) due to septic shock caused by bacterial translocation during treatment for HUS. He showed features of hemolysis, with elevated lactate dehydrogenase (LDH), fragmented red blood cells, and low haptoglobin levels. Plasma free hemoglobin was measured daily throughout the whole course of ECMO with levels higher than 10 mg/dL but not exceeding 50 mg/dL. The extracorporeal membrane oxygenation (ECMO) circuit pressures were carefully monitored to ensure the pump generated no excessive negative pressure. The patient was weaned off ECMO on the eleventh day. There have been several cases of VA-ECMO in patients with HUS; however, there is limited literature on VV-ECMO. As the days on VV-ECMO tend to be longer than those on VA-ECMO, features of hemolysis may complicate management. Although HUS did not directly influence the clinical course in the present case, features of hemolysis were continuously observed. This case highlighted the importance of standard ECMO monitoring, especially daily measurement of plasma free hemoglobin.

## Introduction

Hemolytic uremic syndrome (HUS) is characterized by thrombocytopenia, nonimmune microangiopathic hemolytic anemia, and acute renal failure and is most frequently associated with infections caused by Shiga-like toxin-producing bacteria [1]. Veno-venous extracorporeal membrane oxygenation (VV-ECMO) is a rescue therapy for patients with severe respiratory failure, which does not improve with conventional mechanical ventilation. One of the complications associated with extracorporeal membrane oxygenation (ECMO) is hemolysis due to various factors related to the ECMO circuit, such as negative pressure generated by the pump, blood clots, and excessive centrifugal pump speed. The elevation of plasma free hemoglobin in these patients is related to organ damage and exacerbated morbidity and mortality. A pump exchange is sometimes considered when pump malfunction in indicated. Here, we report a rare case of a patient with HUS and acute respiratory distress syndrome (ARDS) due to septic shock from bacterial translocation requiring VV-ECMO management, with particular attention to hemolytic features throughout the course.

## Case report

The patient was a 26-year-old man with a history of cerebral palsy and epilepsy. He was admitted to a local hospital with a fever and bloody diarrhea. Stool culture testing detected Enterohemorrhagic *E. coli*, indicating a gastrointestinal infection. On the sixth day of hospitalization, blood tests revealed thrombocytopenia, hemolytic anemia, and renal failure. Based on these features, the patient was diagnosed with HUS caused by Shiga toxin-producing *Escherichia coli* (STEC). His blood pressure and oxygenation began to deteriorate on the tenth day of hospitalization, and he was transferred to our hospital.

**Fig. 1:**
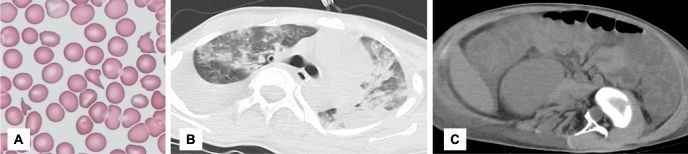
(**a**) Fragmented red blood cells are found in peripheral blood smears. (**b**) Chest CT showing diffuse bilateral ground-glass opacity, consolidation, and interlobular septal thickening. (**c**) Abdomen CT showing intestinal wall thickening and ascites

On arrival, he presented with septic shock, with a systolic blood pressure of 50 mmHg, tachycardia, and metabolic acidosis. Soon after admission to the intensive care unit (ICU), his systolic blood pressure dropped to 26 mmHg, and he went into cardiac arrest. The patient was soon resuscitated and commenced vasopressin and continuous renal replacement therapy (CRRT). Laboratory tests showed features of disseminated intravascular coagulation (DIC) with a platelet count of 20 × 10^9^/L, activated partial thromboplastin time 85.5 s, prothrombin time – international normalized ratio of 1.77, and D-dimer of 6,750 ng/mL. Other laboratory tests showed C-reactive protein (CRP) 427.6 mg/L, white blood cell count 18.83 × 10^9^/L, procalcitonin 0.38 μg/L, hemoglobin 43 g/L, lactate dehydrogenase (LDH) 1478 IU/L, and fragmented red blood cells in the peripheral blood smear (Fig. 1a). The haptoglobin level was 0.441 g/L on arrival to the hospital, which was higher than in the previous hospital, but decreased again to 0.07 g/L on the fourth day of hospitalization. The patient also presented with oliguria and an elevated creatinine of 304.98 μmol/L. ADAMTS13 activity was 31.58%, and ADAMTS13 inhibitor level was below 0.5 BU/mL, excluding thrombotic thrombocytopenic purpura (TTP). AmpC beta-lactamase-producing *Enterobacter ludwigii* was isolated from blood cultures, and the initial antibiotics meropenem and vancomycin were continued. Chest computed tomography (CT) revealed diffuse bilateral ground-glass opacities, consolidation, and interlobular septal thickening, indicating pulmonary edema (Fig. 1b). Abdominal computed tomography (CT) revealed intestinal wall thickening and ascites, indicating severe colitis (Fig. 1c). Based on these findings, we suspected ARDS secondary to septic shock caused by bacterial translocation.

After resuscitation, he required 0.1 µg/kg/minute intravenous infusion of norepinephrine and 0.03 units/minute intravenous infusion of vasopressin to achieve a mean blood pressure of 65 mmHg. He had severe hypoxia with a PaO_2_/F_I_O_2_ ratio of 55 under PEEP settings of 15 cmH_2_O and F_I_O_2_ of 1.0. Arterial blood gas analysis showed pH 7.121, partial pressure of carbon dioxide (PaCO_2_) 51.9 mmHg, bicarbonate (HCO3^−^) 16.5 mmol/L, and lactate 2.7 mmol/L. We performed prone positioning for a few hours but with no oxygenation improvement. Although the patient was an adult, we consulted a pediatric cardiac surgeon for ECMO cannulation because of the short stature and small size of the blood vessels. Since the patient’s respiratory status continued to decline despite high mechanical ventilation settings and prone positioning, and also because the pediatric cardiac surgeon team was needed for ECMO cannulation, the decision to implement VV-ECMO was made on the second day of hospitalization before a full session of prone positioning was performed. Since his blood pressure was manageable with vasopressor drugs, we planned to start with VV-ECMO and insert an additional arterial cannulae when there was prominent hemodynamic instability. A 22 Fr drainage cannula was inserted into the right femoral vein, and an 18 Fr return cannula was placed in the right internal jugular vein. Heparin infusion was used for anticoagulation, along with nafamostat mesylate as an anticoagulant for CRRT to prevent membrane occlusion. As nafamostat mesylate has an extremely short half-life, we used both anticoagulants so that systemic heparin could be titrated according to ECMO anticoagulation protocols while maintaining CRRT anticoagulation and preventing circuit clotting events. Adequate oxygenation was achieved at a flow of 3.3 L/minute, 2900 revolutions per minute (rpm) under lung-protective ventilation settings. His blood pressure gradually improved upon the implementation of ECMO.

**Fig. 2:**
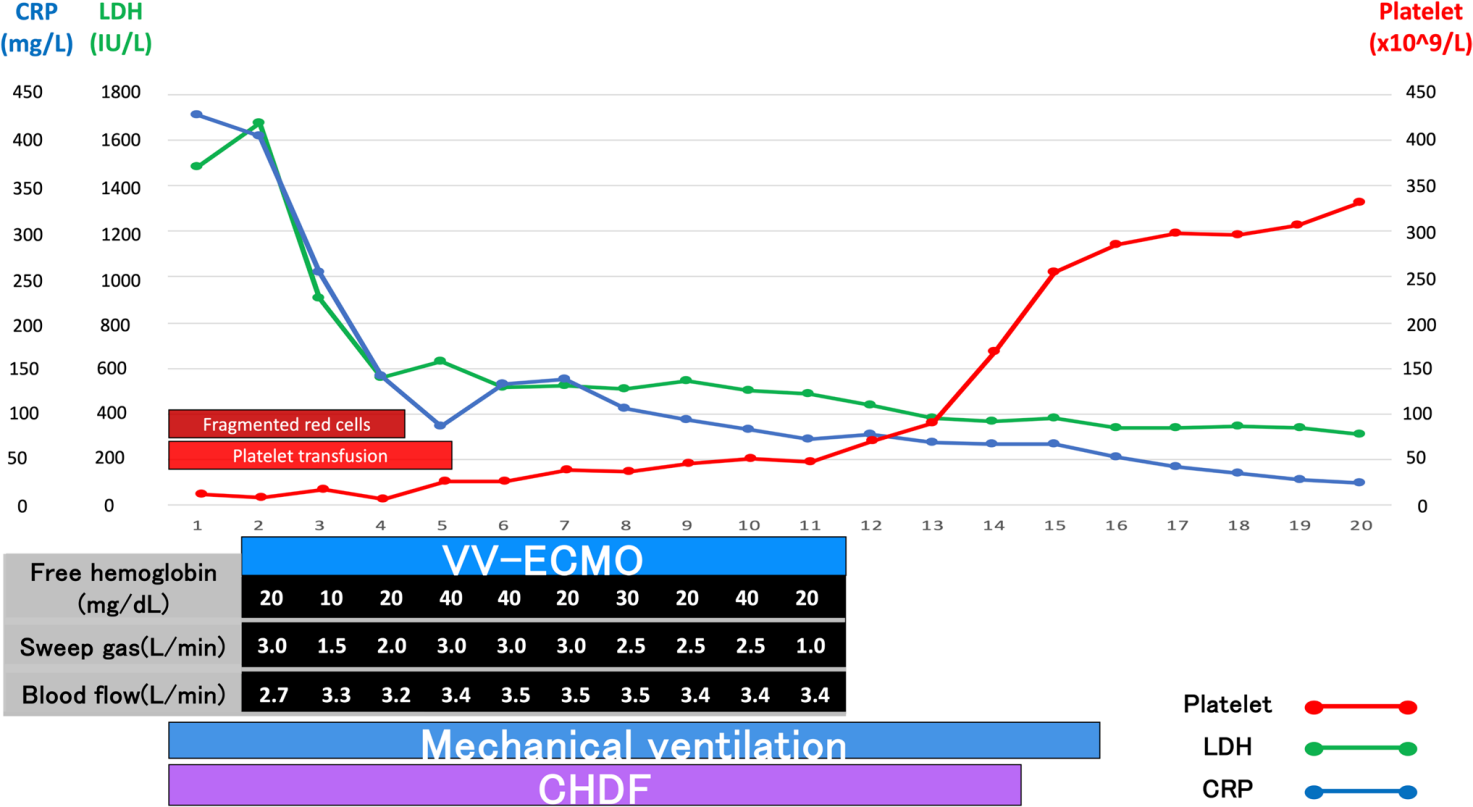
The plasma-free hemoglobin level was higher than 10 mg/dL throughout ECMO but did not exceed 50 mg/dL. LDH and CRP levels rapidly decreased within the first 3, 4 days. Fragmented red blood cells appeared only in the first 4 days of hospitalization

Since features of hemolysis were observed from the beginning of ECMO implementation, careful monitoring was continued. On day one of VV-ECMO, the patient’s plasma free hemoglobin level was 20 mg/dL. Plasma-free hemoglobin was measured daily, with the highest value being 40 mg/dL. However, there were no sudden or pronounced elevations throughout the course (Fig. 2). Other hemolytic features were also observed after the implementation of ECMO. High levels of LDH and fragmented red blood cells were present for the first three days of ECMO but rapidly decreased. Haptoglobin levels increased compared to the level before transfer on the first day of hospitalization but decreased again after the initiation of ECMO. Routine ECMO circuit monitoring of pre-membrane pressure, post-membrane pressure, and pump inlet pressure was performed, along with pre- and post- ECMO membrane blood gas measurement, and anticoagulation monitoring using thromboelastography. While there were prominent features of hemolysis, monitoring of ECMO circuit pressures and oxygenation performance showed no suspicion of thrombosis. Inflammatory markers such as CRP showed marked improvement after hospitalization. The hypoxemia improved, and the patient was weaned off ECMO on the eleventh day of hospitalization without any complications. The patient was weaned off the mechanical ventilator on the fifteenth day and returned to the local hospital on the twenty-second day.

## Discussion

This case demonstrates that VV-ECMO can be successfully used in patients with hemolysis due to medical conditions. Although HUS did not directly influence the clinical course of ARDS, features of hemolysis was continuously present after implementation of ECMO, requiring thoughtful monitoring. The present case highlights the importance of standard ECMO management, particularly daily plasma free hemoglobin measurement along with careful observation of the clinical course of HUS. While plasma free hemoglobin levels were higher than 10 mg/dL throughout the 10 days of ECMO, they did not exceed 50 mg/dL, the level at which the Extracorporeal Life Support Organization (ELSO) guidelines recommend that the cause should be investigated [2]. Other features of hemolysis, such as LDH and fragmented red blood cells was observed during the first three days of ECMO implementation.

While numerous studies show the efficacy of VA-ECMO in patients with HUS complicated by cardiac failure [3, 4], there is limited literature on HUS patients on VV-ECMO. Previous studies show that hemolysis due to ECMO circuit malfunction is higher in VV-ECMO compared to VA-ECMO. One study showed that hemolysis caused by pump-head thrombosis was higher in patients on VV-ECMO [5]. The same study showed that VA-ECMO showed a higher hemolysis rate than VV-ECMO, but this was attributable to premedical treatment such as extracorporeal cardiopulmonary resuscitation (ECPR) or cardiac surgery. Furthermore, the number of days on VV-ECMO tended to be longer than that on VA-ECMO, which may increase the risk of complications associated with ECMO. As hemolysis and elevation of plasma free hemoglobin during VV-ECMO is associated with organ failure and worse morbidity and mortality, it is vital to monitor plasma free hemoglobin and identify the cause. However, determining the cause of elevated plasma free hemoglobin levels may be complicated when there is a pre-existing hemolytic disorder.

In the present case, the plasma free hemoglobin level was higher than 10 mg/dL but did not exceed 50 mg/dL even though other laboratory tests showed signs of hemolysis. Since the hemoglobin molecule is composed of hemes and αβ dimers to form a tetramer in the red blood cell, the hemoglobin can only circulate in the blood briefly with a short half-life of a few hours [6]. Furthermore, when hemoglobin is released into the blood circulation, it is rapidly cleared from the kidney or binds to haptoglobin and cleared from the liver [7]. This may explain the difference between plasma free hemoglobin and other features of hemolysis such as LDH and fragmented red blood cells, indicating that there was no acute ongoing hemolysis at the time of measurement. A sudden pronounced increase in plasma free hemoglobin may indicate pump head thrombosis, in which pump head replacement may normalize levels [8, 9]. In the present case, daily monitoring of plasma free hemoglobin was performed to avoid missing sudden elevations.

Although the time to recovery may vary depending on the severity of HUS, one study showed that the median duration of illness was seven days (range 3–31 days) [10]. In the present case, hemolysis markers, such as LDH and fragmented red blood cells, decreased within the fourth day of hospitalization. Since the patient had already been diagnosed and treated for HUS before respiratory deterioration, it is plausible to think that the clinical course of HUS was toward recovery. Studies have shown that significantly increased plasma free hemoglobin as high as 21.2 µM, was found in patients with Shiga toxin-producing *Escherichia coli* -associated hemolytic uremic syndrome (STEC-HUS) [11]. However, how these levels correlate with disease severity or how rapidly they improve is unknown. Evaluation of the clinical course of HUS is vital to distinguish hemolysis from coexisting disorders or malfunctions of the ECMO circuit.

## Conclusion

A patient with hemolytic features due to HUS was successfully managed with VV-ECMO for ARDS caused by septic shock and bacterial translocation. Although the plasma free hemoglobin level was higher than normal throughout ECMO, daily monitoring of plasma free hemoglobin and other laboratory tests was useful in managing the patient.

## Data Availability

The data for this study are available from the corresponding author on reasonable request.
